# DEVELOPING A HEALTH PROMOTION PROGRAM WITHIN THE CARE LIVING LAB FOR COMMUNITY-DWELLING OLDER ADULTS

**DOI:** 10.1093/geroni/igae098.3340

**Published:** 2024-12-31

**Authors:** Ji Yeon Lee, Eunhee Cho, Jiyoung Shin, Hyeonmi Cho, Bada Kang

**Affiliations:** Inha University, Incheon, Republic of Korea; Yonsei University, Seoul, Republic of Korea; Yonsei University, Seoul, Republic of Korea; Yonsei University, Seoul, Republic of Korea; Yonsei University, Seoul, Republic of Korea

## Abstract

Health promotion for community-dwelling older adults is necessary to facilitate aging in place as the world moves towards an aging society. Substantial efforts have been made to increase the participation of older adults in health promotion programs; however, they were often fragmented due to a lack of active participation of older adults and stakeholders in community settings. This project aimed to co-create a health promotion program for older adults in the community within a care living lab focusing on the five principles: co-creation, engaging end-users and stakeholders, multiple methods, and real-life settings. Older adults as end-users and stakeholders were included in the co-creation of the health promotion program. Multiple methods, such as workshops, meetings, and interviews, were conducted within the existing community program. In total, 37 older adults, 19 frontline staff members, and stakeholders engaged in the co-creation process. The health promotion program ultimately included education for dementia, insomnia, and falls, cognitive worksheets, and exercise, which improved the level of frailty in older adults. The developed program was applied to another group of older adults, effectively improving their physical and cognitive functions, as well as their quality of life. Satisfaction with the program was high, and frontline staff reported positive experiences with the co-creation process. The health promotion program, developed within the care living lab, improved older adults’ health status and brought positive experiences among both older adults and staff. The living lab can be an effective method to improve older adults’ health status by facilitating co-creation and engagement.

